# Revisiting the debriefing debate: does psychological debriefing reduce PTSD symptomology following work-related trauma? A meta-analysis

**DOI:** 10.3389/fpsyg.2023.1248924

**Published:** 2023-12-21

**Authors:** Harry M. Stileman, Christopher A. Jones

**Affiliations:** ^1^Centre of Applied Psychology, University of Birmingham, Birmingham, United Kingdom; ^2^Birmingham and Solihull Mental Health NHS Foundation Trust, Birmingham, United Kingdom

**Keywords:** psychological debriefing, critical incident stress debriefing, early intervention, trauma, PTSD, work

## Abstract

Psychological debriefing is an early post-trauma intervention which aims to prevent the development of PTSD and accelerate normal recovery through discussing, validating, and normalising group members responses to trauma. While originally designed in the 1980s for groups of emergency service personnel, the scope of psychological debriefing extended to individual primary victims of trauma. A Cochrane review in 2002 concluded that psychological debriefing was ineffective, yet some authors have argued that many of the studies that informed the Cochrane review did not adhere to key elements of psychological debriefing. This meta-analysis sought to re-examine the effectiveness of psychological debriefing in preventing or reducing PTSD symptoms following work-related trauma. Appropriate studies were selected from three databases (MEDLINE, Embase and PsycINFO). Inclusion criteria was intentionally broad so that features of psychological debriefing that may determine its effectiveness could be explored through a series of subgroup analyses. The overall synthesis did not find consistent evidence that psychological debriefing helps to prevent or reduce PTSD symptoms following work-related trauma. Shortcomings in the methodology and reporting of many of the studies meant that several important subgroup analyses could not be conducted. Further well-designed studies in this field are warranted to ensure that employees exposed to potentially traumatic events receive the effective support they need and deserve.

## Introduction

Occupational groups such as military personnel, emergency service workers and healthcare workers are routinely exposed to potentially traumatic events (PTEs), increasing their risk of developing mental health difficulties such as post-traumatic stress disorder (PTSD; Skogstad et al., 2013; Petereit-Haack et al., 2020). The World Health Organisation’s *International Classification of Diseases* 11th Revision (ICD-11; WHO, 2018) notes that PTSD “may develop following exposure to an extremely threatening or horrific event or series of events” (ICD-11; WHO, 2018) and consists of three clusters of symptoms: (1) re-experiencing of the trauma through intrusive memories, flashbacks and nightmares, (2) avoidance of reminders of the trauma, and (3) hyperarousal and hyperreactivity associated with the traumatic event.

PTSD and other trauma-related mental health difficulties can have far-reaching consequences for the individual, including adverse effects upon health, productivity at work and the quality of relationships with those close to them (Brooks et al., 2019; Lee et al., 2020). It is therefore important that organisations in which the likelihood of exposure to trauma is high have effective management strategies in place to support their employees. This is both a moral responsibility and a legal obligation. The Health and Safety at Work Act (1974) states that employers have a duty of care “to ensure, so far as is reasonably practicable, the health, safety and welfare at work of all employees” (p. 4). One management strategy that has been widely used for decades is “psychological debriefing”.

Psychological debriefing has its origins in World War I (Litz et al., 2002). Following a battle, commanders would “debrief” their soldiers. The rationale was that sharing stories would help boost the morale of soldiers and prepare them for future conflict. Military psychiatrists also developed strategies to support soldiers who were experiencing traumatic stress reactions. Underlying these strategies were the principles of proximity, immediacy, and expectancy (Grinker and Spiegel, 1944). Soldiers were supported near the battlefield, soon after the onset of difficulties, and with the expectation of a quick return to combat.

In the 1980s, a psychologist and former firefighter called Jeffrey Mitchell noted similarities between the stress of combat and the stress of emergency services and developed the most widely used method of psychological debriefing - Critical Incident Stress Debriefing (CISD) - as part of his Critical Incident Stress Management Programme (Mitchell, 1983). CISD is a seven phase intervention which was specifically designed for groups of emergency service workers following exposure to a PTE, or what Mitchell termed a ‘critical incident’. Mitchell went on to collaborate with another psychologist, Atle Dyregrov, who developed a seven phase model similar to CISD and coined the alternative term Psychological Debriefing (Dyregrov, 1989). The term “psychological debriefing” will be used to refer collectively to these two models hereon in.

Psychological debriefing aims to prevent the development of PTSD and accelerate normal recovery through discussing, validating, and normalising group members responses to trauma (Mitchell and Everly, 1996). This aim is in keeping with the cognitive model of PTSD (Ehlers and Clark, 2000) which proposes that misconceptions and negative appraisals relating to a traumatic event and its sequalae play a role in the development and maintenance of PTSD symptoms. Further aims of psychological debriefing include enhancing group cohesion, providing information about coping strategies, screening for individuals who need further support and referring on for further assessment or intervention if required (Mitchell and Everly, 1996).

Psychological debriefings as described by Mitchell and Everly (1996) are typically led by two facilitators, although for larger groups there can be up to four facilitators. Facilitators should include a mental health professional and a specially trained peer support worker from the same profession as the group members. Debriefings usually involve a single session, lasting between 1 and 3 h. They are typically facilitated 24 to 72 h after the PTE, although significant delays can often occur.

Following Mitchell’s (1983) seminal paper, the scope of psychological debriefing extended beyond groups of emergency service personnel to other occupations, including the military and healthcare. Furthermore, it was employed for individual primary traumas outside of an occupational setting, including burns (Bisson et al., 1997), violent crime (Rose et al., 1999), childbirth (Priest et al., 2003) and road traffic accidents (Hobbs et al., 1996).

In 2002, the Cochrane Collaboration for Evidence-based Practice published a review of the effectiveness of single-session psychological debriefing in preventing PTSD, which was updated in 2010 (Rose et al., 2002). Fifteen randomised controlled trials met inclusion criteria. No consistent and substantive evidence was found that psychological debriefing reduces the risk of developing PTSD symptoms compared to no intervention and two trials which included longer follow up periods (Hobbs et al., 1996; Bisson et al., 1997) reported adverse effects. Consequently, Rose et al. (2002) concluded that “psychological debriefing is either equivalent to, or worse than, control or educational interventions in preventing or reducing the severity of PTSD” (p. 2).

As a result of this Cochrane review (Rose et al., 2002), the National Institute for Health and Care Excellence (NICE) completed its own systematic review of seven RCTs in this field which consisted of many of the same studies as the Cochrane review, including both the studies by Bisson et al. (1997) and Hobbs et al. (1996). It also concluded that “single-session debriefing may be at best ineffective” (NICE, 2005, p. 84).

NICE guidance for PTSD has since been unequivocal in its recommendation to “not offer psychologically-focused debriefing for the prevention or treatment of PTSD” (National Institute for Health and Care Excellence, 2018, p. 15). Consequently, organisations have been left with limited guidance on suitable strategies to maintain the psychological wellbeing of their staff following exposure to PTEs. In some organisations, the provision of psychological debriefing for PTEs was withdrawn (Hawker and Hawker, 2015). In other organisations, psychological debriefing continues to be offered, sometimes under different names (e.g., “Powerful Event Group Support;” Hawker et al., 2011). The United Kingdom military now use Trauma Risk Management (TRiM), which shares many of the same objectives and practices as psychological debriefing (Greenberg et al., 2008). TRiM is a peer-support system which aims to ensure that employees exposed to trauma are properly supported. TRiM provides information, assesses risk of trauma reactions and signposts for support if psychological distress does not resolve spontaneously (Whybrow et al., 2015). Other organisations offer non-specific interventions such as “psychological first aid” (PFA), which broadly involves the provision of information, comfort, emotional care and practical support (Shultz and Forbes, 2014). Unlike psychological debriefing, which was designed for groups of workers exposed to trauma, PFA was originally intended for use with individuals across the lifespan who were exposed to disaster or terrorism. PFA shares many components with psychological debriefing but generally avoids any recounting of an individual’s perceptions and emotional reactions during a stressful event. While this approach is promoted in practice guidelines (Inter-Agency Standing Committee, 2007; World Health Organization, 2011), there is currently a lack of evidence of its effectiveness (Dieltjens et al., 2014).

As with psychological debriefing itself, the Cochrane review of psychological debriefing (Rose et al., 2002) has provoked controversy. Two independent review papers (Hawker et al., 2011; Tamrakar et al., 2019) note some of the alternative explanations for the two negative outcomes reported by Hobbs et al. (1996) and Bisson et al. (1997). Firstly, debriefed participants had been more severely injured than those who were not debriefed. When this was controlled for, the negative outcomes of debriefing of trauma symptoms were either eliminated (Bisson et al., 1997) or reduced to marginal significance (Hobbs et al., 1996; Mayou et al., 2000).

Secondly, the scope and nature of the interventions evaluated by these two RCTs were inconsistent with key features of psychological debriefing: some of the debriefings were too short (under an hour); the facilitators often lacked adequate training; debriefings included a detailed review of the PTE rather than a brief overview; and the participants were individual victims of trauma among the general public, rather than groups of professionals for whom the intervention was originally developed. This was recognised by the follow-up review by NICE, which stated that “no trial on critical incident stress debriefing as it was originally conceived by Mitchell and colleagues (i.e., as a group intervention for teams of emergency workers, military personnel or others who are used to working together)… met our methodological inclusion criteria” (NICE, 2005, p. 84).

These criticisms of the Cochrane review (Rose et al., 2002) have led to more recent suggestions that psychological debriefing may have been dismissed too quickly and calls for further investigation to clarify the potential benefits of psychological debriefing (Tamrakar et al., 2019; O’Toole and Eppich, 2022). Hawker and Hawker (2015) outline four lessons that can be learnt from the Cochrane review findings: (1) do not offer debriefing too soon after a traumatic event; (2) do not offer debriefing lasting less that 1 h; (3) do not use insufficiently trained or inappropriate facilitators; (4) do not probe too hard for details.

A scoping review was recently undertaken by Public Health England’s Behavioural Science Research Team (Richins et al., 2020) to identify research evaluating early interventions in occupations in which there is a high risk of exposure to PTEs. The review included 50 studies of mixed quality and method and included both quantitative and qualitative data. Qualitative outcomes were assessed using meta-ethnography. However, a meta-analysis was not conducted which is likely because of the wide range of interventions included within the review such as exposure therapy, cognitive behavioural therapy and compassion focused therapy in addition to psychological debriefing. Nevertheless, most of the interventions included within the review were based on psychological debriefing and Richins et al. (2020) note that most of these led to a reduction in symptom severity. Furthermore, in the 12 studies where severity scores did not change, half were still evaluated as being helpful by the participants. Richins et al. (2020) concluded that psychological debriefing can be an effective support in emergency responders (for which psychological debriefing was originally intended) when they adhere to key components of established models and are: (a) informed by the organisational culture, (b) have the support of management, and (c) utilise existing peer support systems within teams.

This meta-analysis aims to examine the evidence-base into the effectiveness of psychological debriefing in preventing or reducing PTSD symptoms following work-related PTEs. In contrast to the Cochrane review of psychological debriefing (Rose et al., 2002), this review extended the scope of studies beyond RCTs to include other non-randomised or uncontrolled designs. The rationale for this came from the recognition that there are implicit difficulties in conducting methodologically robust RCTs when evaluating psychological debriefing (Deahl, 2000). Trauma generally occurs in unpredictable and chaotic circumstances. As a result, researchers are often required to work opportunistically within strict time constraints and in line with operational processes. Furthermore, there are ethical dilemmas with employing randomised non-intervention controls for participants who may want, and benefit from, psychological debriefing. Consequently, a lot of the research on the effectiveness of psychological debriefing would not meet the criteria insisted upon by the Cochrane Library.

Due to insufficient studies within the Cochrane review of psychological debriefing (Rose et al., 2002), formal subgroup analysis was not undertaken to explore potential sources of heterogeneity in outcomes. Through including a wider range of study designs, this study sought to utilise subgroup analyses to identify key components that may determine the effectiveness of psychological debriefing, including those proposed by Hawker and Hawker (2015) and Richins et al. (2020) such fidelity to an established model, the length of debriefs and the extent of the debriefers training.

## Methods

### Search strategy

#### Inclusion criteria

Full inclusion and exclusion criteria are described in Table 1.

**Table 1:** Inclusion and exclusion criteria.

Inclusion criteria	Justification
*Nature of intervention*	
Studies that have referred to their intervention as a ‘debriefing’ and involve some recollection of the trauma and subsequent reactions.	While there are a range of different terms to refer to psychological debriefing (e.g., stress debriefing, critical incident stress debriefing, crisis intervention), to ensure internal validity of the meta-analysis it was important that there is homogeneity between the content of psychological debriefings included in this review.
Exclude: psychological therapies (e.g., CBT, EMDR, CFT).	These therapies are outside of the scope of this review.
*Participant characteristics*	
Employees who have experienced a work-related traumatic event.	Psychological debriefing was originally intended for work-related trauma, and this remains the scope of this review.
*Outcome data*	
Studies include a measure of PTSD symptoms.	To ensure internal validity of the meta-analysis, only studies with validated measures of PTSD symptoms (either self-report or structured assessment) were included.
The studies are required to report either means and standard deviations, or F- Test statistics, or Cohen’s *d* effect size.	This was to ensure that outcomes can be calculated into an effect size for the purpose of the meta-analysis.
*Type of article*	
Studies published in English language.	English is the first language of the authors.
Articles published in peer-reviewed journals.	This was to ensure methodological rigour in the articles included.
The following article types were excluded: meta-analysis, reviews, theoretical pieces, commentaries, clinical guidance, study protocols, opinion pieces.	These articles do not provide the outcome data needed for this meta-analysis.
*Study design*	
The following study designs were excluded: single-case designs, case series, samples where *n* < 10.	This was to ensure that an effect size reported by the included studies could be calculated with methodological rigour. While previous reviews in this area have only include randomised controlled trials (Rose et al., 2002), it was recognised that RCTs represent only a small proportion of the research evidence and so a broader range of study designs were included.

#### Search of electronic databases

A systematic search of the literature was carried out on 14th November 2023 using MEDLINE, Embase and PsycINFO. The aim of the search was to obtain a comprehensive overview of the literature into the effectiveness of psychological debriefing in preventing the development of trauma reactions in individuals exposed to work-related PTEs. The search terms that were used to identify these studies are outlined.

### Generic search terms

(Early adj3 intervention* ‘OR’ debrief* ‘OR’ psychological intervention ‘OR’ crisis intervention ‘OR’ critical incident stress debrief* ‘OR’ critical incident stress management) ‘AND’ (PTSD ‘OR’ posttrauma* ‘OR’ post trauma* ‘OR’ post-trauma* ‘OR’ traumatic stress ‘OR’ stress disorder* ‘OR’).

#### Specific MEDLINE search terms

(Early adj3 intervention* ‘OR’ debrief* ‘OR’ psychological intervention ‘OR’ crisis intervention ‘OR’ critical incident stress debrief* ‘OR’ critical incident stress management) ‘AND’ (stress disorders, traumatic/ or combat disorders/ or psychological trauma/ or stress disorders, post-traumatic/ or stress disorders, traumatic, acute/).

#### Specific PsycINFO search terms

(Early adj3 intervention* ‘OR’ debrief* ‘OR’ psychological intervention ‘OR’ crisis intervention ‘OR’ critical incident stress debrief* ‘OR’ critical incident stress management) ‘AND’ (posttraumatic stress disorder/ or exp. “stress and trauma related disorders”/ or exp. acute stress disorder/ or exp. posttraumatic stress/).

#### Specific Embase search terms

(Early adj3 intervention* ‘OR’ debrief* ‘OR’ psychological intervention ‘OR’ crisis intervention ‘OR’ critical incident stress debrief* ‘OR’ critical incident stress management) ‘AND’ (exp posttraumatic stress disorder/).

### Data extraction and quality assessment procedures

All data was extracted by a single author. It was expected that outcome data would be expressed as a mean, standard deviation, and sample size for each of a psychological debriefing intervention group and a control condition. Where such data was not reported, then effect sizes were calculated from F- or T-tests for these outcomes.

#### Defining problematic variance

As well as reporting a mean effect size, this meta-analysis sought to quantify and analyse the between-study heterogeneity. High levels of heterogeneity may arise between studies due to differences in interventions, participant characteristics, outcome measures or methodology (von Hippel, 2015).

Higgins *I*^2^ (Higgins and Thompson, 2002) is a commonly used statistic to measure to amount of dispersion between studies. It is expressed as a percentage (0 to 100%) and provides an indication of the proportion of variation which is attributable to between-study variance rather than differences in precision of measurement due to sample size differences. In line with the benchmarks set by Higgins et al. (2003) and recognising the considerable variation in methodologies of the primary studies included within the synthesis, problematically high heterogeneity was defined as a Higgins *I*^2^ value of more than 75%. Where problematic heterogeneity was observed, analyses were conducted to identify the source of heterogeneity between the effect sizes of the primary studies.

While standardised effect sizes from both repeated measures and independent-groups designs can be combined in a meta-analysis (Borenstein et al., 2009), it must be determined that potential sources of bias are not impacting the effect size estimates of certain study designs (Morris and DeShon, 2002). Consequently, a subgroup analysis was conducted to determine whether these study outcomes differed in substantive ways.

#### Risk of bias assessment

A study hierarchy was implemented to assess the contribution of each of the study designs to the overall quality score (see Supplementary Table 1). A set of quality criteria were developed to assess any risk of bias within this literature. The quality criteria were adapted from existing risk of bias frameworks, particularly The Cochrane Collaboration Risk of Bias Tool (Higgins et al., 2011) and the Risk of Bias Assessment Tool for Nonrandomised Studies (Kim et al., 2013). Risk of bias was assessed in seven domains: selection bias, performance bias, treatment fidelity, detection bias, statistical bias, reporting bias and generalisation (see Supplementary Table 2).

A quality index score was calculated for all papers included within the meta-analysis. This score was calculated using the study’s overall design as assessed by the study design hierarchy and the risk.

## Results

**Figure 1:**
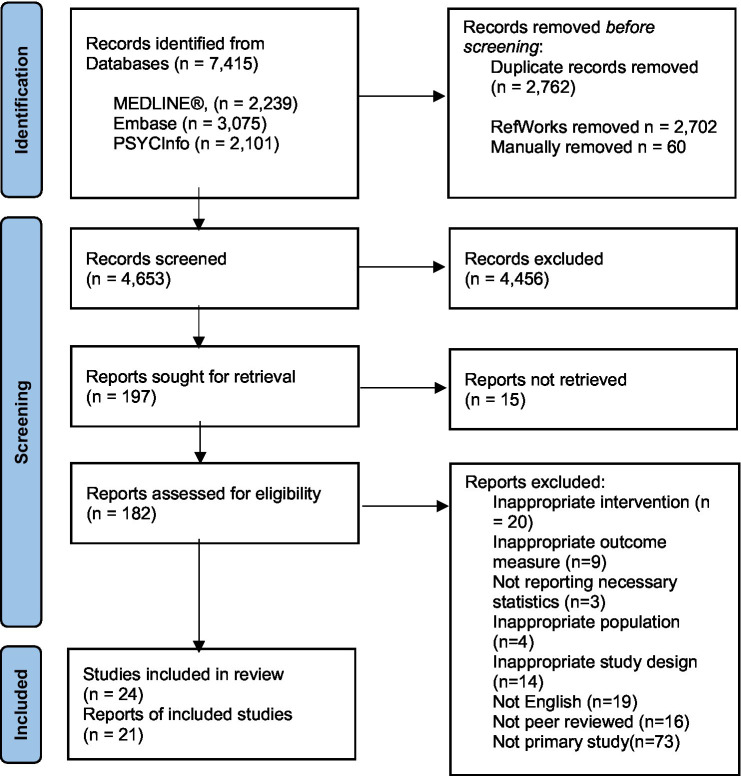
Process of study selection: PRISMA diagram (Page et al., 2021).

The results of the systematic search are presented in Figure 1. The search yielded a total of 7,415 articles and 4,653 once duplications were removed. Sensitivity in the search strategy was privileged over specificity as any further grouping of search terms to narrow articles down to work-related traumas resulted in known papers being lost. The inclusion criteria were used to screen these 4,653 articles by title and abstract. The three most common reasons articles were excluded at this stage were that they either did not relate to psychological debriefing, they did not relate to work-related trauma, or they did not provide outcome data (i.e., review papers). The remaining 197 articles were sought for retrieval; however, it was not possible to retrieve 15 of these articles. The full text of the remaining 182 articles were then reviewed in more detail against the exclusion criteria. Twenty-four articles met the full inclusion criteria. Three articles which met the inclusion criteria could not ultimately be included in the synthesis: Shalev et al. (1998) used a measure of PTSD symptoms pre-intervention but not post-intervention; Söndergaard (2008) included traumatic events that occurred outside of the workplace; and Deahl (2000) did not specify the number of participants who completed the outcome measures.

### Data extraction

Several studies reported group means across multiple timepoints. In these cases, data was extracted for each timepoint. Timepoints were then grouped into the following categories: ‘short-term’ when outcome measures were collected 0–3 months after debriefing; ‘medium-term’ when outcome measures were collected 4–6 months after debriefing; ‘long-term’ when outcome measures were collected 7 months or more after debriefing. In both Kenardy et al. (1996) and Wu et al. (2012), more than one timepoint fitted into the same time category and so only one of these datasets was extracted. When studies had included outcomes from multiple timepoints, unless the impact of time on outcome scores was being directly analysed, scores from the first data collection timepoint following intervention was used in analysis to avoid replication.

Some studies reported PTSD symptom cluster subscale scores on outcome measures (avoidance, hyperarousal, intrusion), while other studies only reported overall scores. Three studies (Carlier et al., 2000; Tehrani et al., 2001; Harris et al., 2002) only reported cluster subscale scores. However, as these subscales included all the items on the PTSD measures used, the total mean score could be calculated and ultimately transformed into an estimate of Cohen’s *d*.

Fifteen studies used independent-group designs and reported means, standard deviations, and sample sizes of both a group who received psychological debriefing and a control group who either received no intervention, lower-level support such as stress education, or were on a waiting list for an intervention. Tehrani et al. (2001) did not report standard deviations for each group so pooled standard deviations were substituted. Carlier et al. (1998) did not report means and standard deviations, instead reporting the percentage of participants in both the experimental and control groups that met a threshold for a PTSD diagnosis. In this case, percentages were converted into log ratios and then into estimates of Cohen’s *d* using the sample sizes reported.

Two studies (Campfield and Hills, 2001; Richards, 2001) included a comparator group rather than a control group, in which participants also received a form of psychological debriefing. In the study by Campfield and Hills (2001), participants either received an “immediate” (<10 h) or “delayed” (>48 h) debriefing. Data for both groups was extracted but treated separately as two before-and-after studies. In the study by Richards (2001), one group received CISD, and the other group received the more extensive package of critical incident stress management. Again, this study was treated as a before-and-after study and only data from the CISD group was extracted. One between-group study (Ruck et al., 2013) reported significantly different baselines scores of PTSD symptoms between the experimental group and control group. As this was not controlled for in the statistical analysis (e.g., by using a treatment x timepoint ANCOVA), this study was also treated as a before-and-after study and only the data from the experimental group was extracted.

Both Matthews (1998) and Carlier et al. (2000) used two independent control groups in their studies. Carlier et al. (2000) included an “external control group” of participants who had experienced trauma before debriefing was introduced in the workplace and an “internal control group” of participants who had declined the offer of debriefing. As a different outcome measure was used with the external control group, only data from the experimental group and internal control group were used. Matthews (1998) included one control group consisting of participants who did not request debriefing and another control group consisting of participants from a different area to the other two groups who did not receive debriefing because it was not available. In this case, both control group outcomes were combined into a single quantitative outcome using the procedure described by Borenstein et al. (2009).

Adler et al. (2009) presented adjusted means and standard deviations comparing the experimental and control groups by combat exposure levels. In this instance, to ensure participants had all been exposed to trauma, data from the top-third exposure level (*n* = 326) was extracted.

### Quality assessment

#### Selection bias

Selection bias was mixed within the studies. Ten studies were rated as low risk of bias due to reasons such as providing clear descriptions of the study population and recruitment methods, finding no significant differences in baseline characteristics between groups and acceptable levels of non-response rates. Five studies were rated as unclear. Four of these studies (Kenardy et al., 1996; Wee et al., 1999; Regehr and Hill, 2001; Harris et al., 2002) adopted a naturalistic design in which they approached participants who had or had not attended a psychological debriefing following a PTE at work retrospectively. As a result, these studies could not discount systematic differences between participants who attended psychological debriefing and those that did not. The remaining eight studies were rated as high-risk of bias, primarily due to clear differences between the groups being compared, including different occupations (Chemtob et al., 1997; Eid et al., 2001; Humphries and Carr, 2001) or different geographical areas (Matthews, 1998). In two studies (Carlier et al., 2000; Ruck et al., 2013), the intervention and control groups were formed through self-selection, with the control group consisting of those who had declined debriefing. As a result of this self-selection, the debriefed groups may have consisted of people more negatively impacted who sought out help (Tuckey, 2007).

#### Performance bias

All studies were rated as unclear risk of performance bias. This was primarily due to the studies being unable to blind participants to the intervention they were receiving. All but two of the studies collected self-report measures of PTSD symptoms. In these cases, participants’ awareness of the intervention they were receiving, rather than the intervention itself, may have influenced their self-reported scores. The remaining two studies were rated as unclear due to a lack of clarity surrounding the information given to participants prior to taking part in the study, meaning that it was not possible to determine whether participants were differentially motivated (Carlier et al., 1998; Wu et al., 2012).

#### Treatment fidelity

Treatment fidelity was mixed within the studies. While most studies reported adhering to a seven phase model of psychological debriefing, only three studies provided evidence of treatment fidelity being appropriately assessed through the independent scoring of protocol adherence (Adler et al., 2008, 2009; Wu et al., 2012). Consequently, all the other studies were rated as either unclear risk or high risk. Six studies were rated as high risk either due to there being no assurances that facilitators were trained in delivering psychological debriefing (Chemtob et al., 1997; Tehrani et al., 2001) or researchers having no control over the intervention provided to participants (Kenardy et al., 1996; Wee et al., 1999; Regehr and Hill, 2001; Harris et al., 2002).

#### Detection bias

The majority of studies were rated as low-risk of detection bias as they used well established outcome measures of PTSD symptoms with good psychometric properties such as the Impact of Event Scale (IES; Horowitz et al., 1979), IES-revised version (Weiss, 2007) or PTSD Checklist (PCL; Weathers et al., 1993) and implemented these measures consistently across participants. In the two studies which used assessor ratings rather than self-rating, these assessors were blinded to the debriefing status of participants (Carlier et al., 1998; Wu et al., 2012). The remaining five studies were rated as unclear risk. In three cases, this was due to the study using a less well-established measure devised by an author of the paper without sufficient justification for this decision (Carlier et al., 2000; Tehrani et al., 2001; Shoval-Zuckerman, 2015). Other reasons for studies being rates as unclear risk were not reporting the psychometric properties of the measures used (Wee et al., 1999) or reporting a total score based on the combination of two separate outcome measures (Matthews, 1998).

#### Statistical bias

Eight studies were rated as low risk for statistical bias, with seven as unclear and six as high. Seven of the eight studies rated as low risk used appropriate statistical testing and reported no data loss, while one had an attrition marginally above 5%, but used intention-to-treat analysis (Grundlingh et al., 2017). Studies were primarily rated as unclear due to a lack of clarity regarding the statistical testing used or attrition rates between 10 and 20%, while the six high-risk studies had attrition rates above 30%.

#### Reporting bias

Overall, the full reporting of the outcome within studies was good, with 19 of the studies being rated as low risk of reporting bias. One study was rated as unclear risk as statistics were not reported for most of the data and, instead, presently solely as percentages (Carlier et al., 1998). One study was rated as high risk as the six-month follow-up data was not reported, with only a statement provided that “no significant difference” was found between the experimental and control groups (Carlier et al., 2000).

#### Generalisability

The majority of studies included within this meta-analysis were looking at the effectiveness of psychological debriefings within a specific occupation and demonstrated no intention to extrapolate these findings outside of this population. Consequently, ratings for generalisability were mostly determined by the sample sizes in studies. Ten studies were rated as low risk, with some of these studies, particularly those in military research, using very high sample sizes (Adler et al., 2008, 2009; Wu et al., 2012). However, the other eleven studies were rated as either unclear or high risk due to the small sample sizes used and no evidence of power analysis being conducted, or other justifications provided, for the sample size utilised.

#### Summary

Overall, there was a mixed level of bias across the 21 studies included in the meta-analysis (see Supplementary Table 3). However, due to the difficulties in conducting randomised controlled trials with trauma, poorer quality studies with medium to high risk of bias were included. Consequently, sensitivity analysis was used to empirically assess the impact of methodological variations.

### Selection of the meta-analytic model

**Figure 2:**
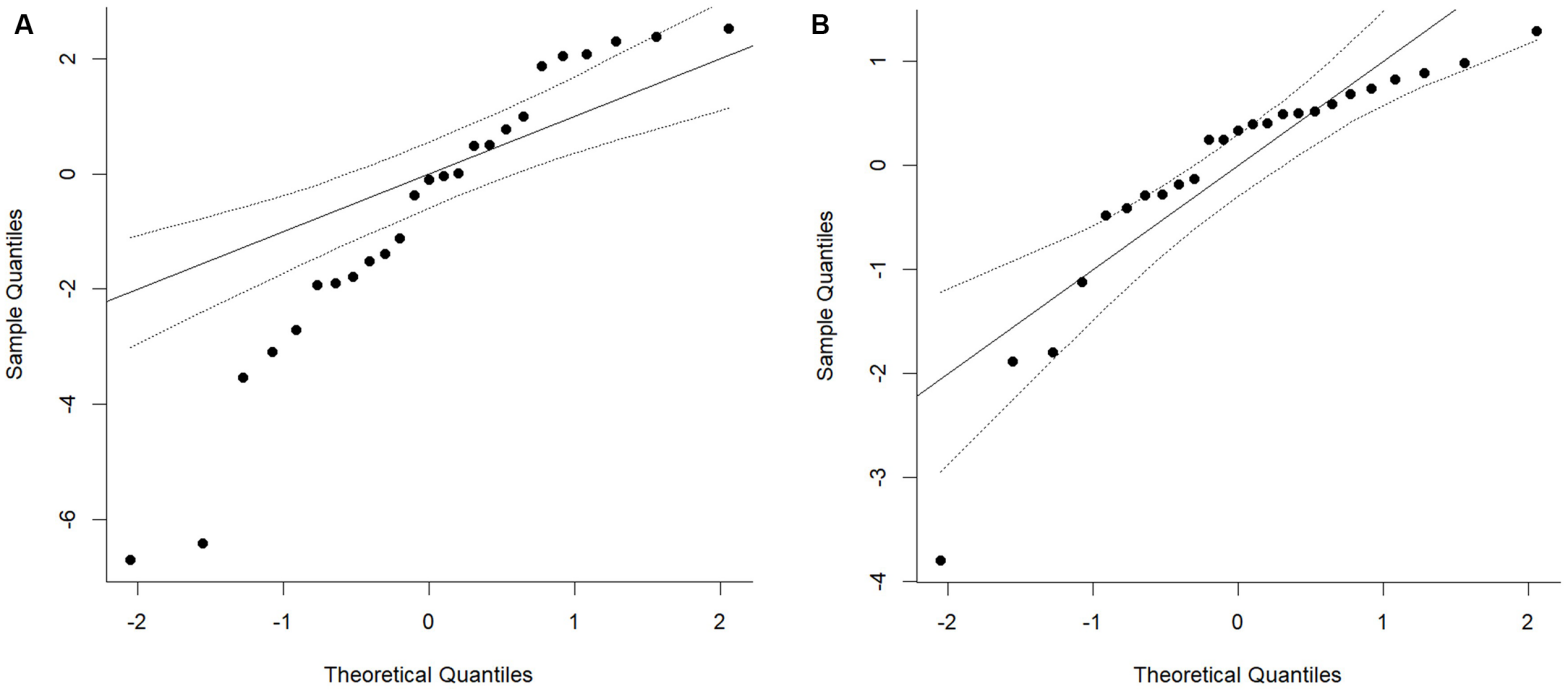
QQ plot of the distribution of standardised mean differences within the primary studies. Chart A shows the fixed effects model and Chart B depicts the random effect model using the restricted maximum-likelihood estimator.

The distribution of primary study effects is shown in Figure 2. The between-study variance (tau^2^) was calculated using the restricted maximum-likelihood estimator (Banks et al., 1985).

As can be seen from Figure 2, the fixed effects model (Chart A) shows clear evidence of non-normality in the distribution of standard mean differences within the primary studies. While the random effects model using the restricted maximum-likelihood estimator also shows some evidence of non-normality, 90% of the primary study effects fall within the 95% confidence intervals for the expected normal values. This indicates that the use of random effect model using the restricted maximum-likelihood estimator estimate was an appropriate method for the calculation of the variation of the true effect.

### Omnibus test of total score on PTSD measures

The standardised mean differences described in the primary studies are reported in Table 2. There were 21 studies reporting a total of 3,744 participants. Participants were recruited from a variety of occupations including military, emergency services, healthcare, prison and care sectors, as well as occupations where there is a lower risk of work-related PTEs such as financial and retail sectors. The reasons for the psychological debriefings taking place were predominantly due to a single, discrete event such as a robbery, assault, or road traffic accident (15 studies). However, for studies using military samples, psychological debriefings were predominantly offered due to multiple PTEs occurring during a deployment. In 18 of the studies, a single debriefing session was offered, with only two studies offering more than one debriefing session (Carlier et al., 2000; Grundlingh et al., 2017) and Kenardy et al. (1996) including in their sample both participants who had attended a single session and those that had attended multiple sessions. Studies took place in a variety of geographical locations including the United Kingdom, United States, Australia, Netherlands, Norway, Uganda, Ireland, Israel, and China. Most of the studies included mixed gender samples, although studies with participants from the military or emergency services consisted of predominantly male or all-male samples.

**Table 2:** Treatment effects reported in the primary studies (using first [or only] data collection time point for each study).

Study name	Year	Cohen’s *d*	*SE*	*N*	Study design	Single or multiple incident	Single or multiple debrief	Area of employment
Campfield and Hills (2001) (delayed debrief)	2001	−0.71	0.32	41	Before-and-after study	Single	Single	Fast food, hotel, petrol service station, rail, video store
Campfield and Hills (2001) (immediate debrief)	2001	−3.89	0.57	36	Before-and-after study	Single	Single	Fast food, hotel, petrol service station, rail, video store
Richards (2001)	2001	−1.86	0.28	75	Before-and-after study	Single	Single	Finance
Ruck et al. (2013)	2013	−0.61	0.28	55	Before-and-after study	Single	Single	Prison staff
Tehrani et al. (2001)	2001	2.17	0.67	12	Before-and-after study	Single	Single	Supermarket
Carlier et al. (1998)	1998	0.00	0.20	105	Non-randomised controlled trial/experiment	Single	Single	Emergency services
Carlier et al. (2000)	2000	0.16	0.14	168	Non-randomised controlled trial/experiment	Single	Multiple	Emergency services
Chemtob et al. (1997)	1997	−1.29	0.34	43	Non-randomised controlled trial/experiment	Single	Single	Disaster workers
Deahl et al. (1994)	1994	−0.19	0.27	62	Non-randomised controlled trial/experiment	Multiple	Single	Military
Eid et al. (2001)	2001	−0.64	0.48	18	Non-randomised controlled trial/experiment	Single	Single	Military and emergency services
Harris et al. (2002)	2002	0.04	0.07	660	Non-randomised controlled trial/experiment	Single	Single	Emergency services
Humphries and Carr (2001)	2001	−0.79	0.39	34	Non-randomised controlled trial/experiment	Single	Single	Finance, retail, hospital emergency
Kenardy et al. (1996)	1996	0.22	0.15	195	Non-randomised controlled trial/experiment	Single	Both	Emergency services and disaster workers
Matthews (1998)	1998	−0.12	0.30	63	Non-randomised controlled trial/experiment	Single	Single	Care workers
Regehr and Hill (2001)	2000	0.35	0.20	127	Non-randomised controlled trial/experiment	Single	Single	Emergency services
Shoval-Zuckerman (2015)	2015	−0.52	0.16	166	Non-randomised controlled trial/experiment	Multiple	Single	Military
Wee et al. (1999)	1999	−0.49	0.26	65	Non-randomised controlled trial/experiment	Multiple	Single	Emergency services
Adler et al. (2008)	2008	−0.10	0.10	382	Randomised controlled trial/experiment	Multiple	Single	Military
Adler et al. (2009)	2009	−0.21	0.09	514	Randomised controlled trial/experiment	Multiple	Single	Military
Grundlingh et al. (2017)	2017	0.62	0.28	52	Randomised controlled trial/experiment	Multiple	Multiple	Violence researchers
Tuckey and Scott (2014)	2014	0.15	0.32	39	Randomised controlled trial/experiment	Single	Single	Emergency services
Wu et al. (2012)	2012	−0.03	0.07	832	Randomised controlled trial/experiment	Single	Single	Military

### The impact of study design on effect size

**Figure 3:**
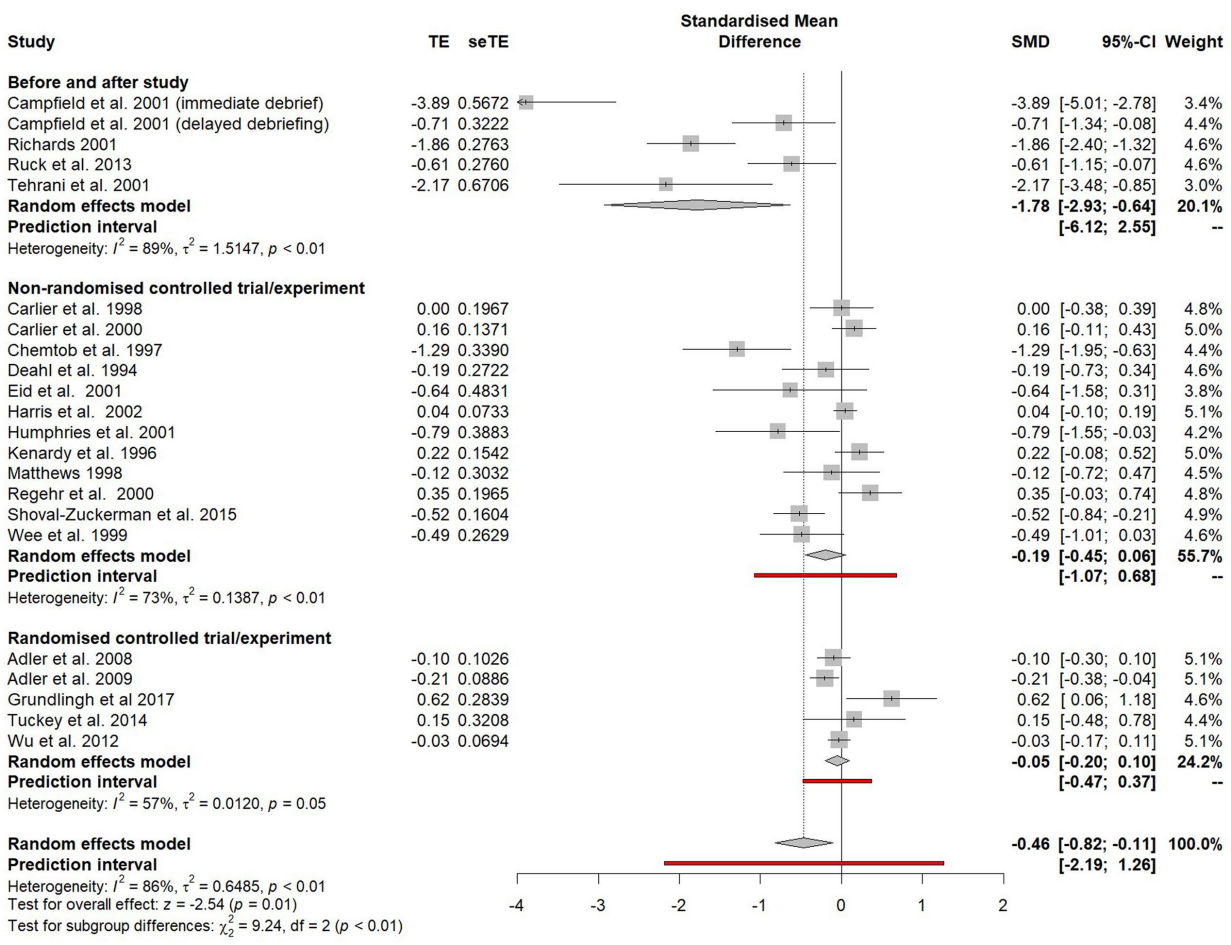
Subgroup plot on the impact of study design on estimated effect size.

A random effects models was calculated using the generic inverse variance method to compare the effect size estimates of the three different study designs included within the meta-analysis (see Figure 3). The weighted average standardised mean difference for before-and-after studies (SMD = −1.78, 95% CI = 2.93 to −0.64) was significantly different (*χ*^2^ = 9.24, *p* < 0.01) to the SMD for both non-randomised controlled trials (SMD = −0.19, 95% CI = −0.45 to 0.06) and randomised controlled trials (SMD = −0.05, CI = −0.20 to 0.10). The magnitude of the effect size estimate in the before-and-after studies is likely to have been inflated by the maturational biases inherent in this study design. Consequently, all uncontrolled before-and-after studies were removed from the meta-analysis.

There was no significant difference (*χ*^2^ = 0.94, *p* = 0.33) between the effect size estimates of non-randomised controlled trials and randomised controlled trials, so these study designs were combined for the subsequent analyses. Furthermore, when before-and-after studies were excluded, heterogeneity went from being unacceptably high (*I*^2^ = 86%) to below the 75% threshold (*I*^2^ = 69%) and so a ‘leave-one-out’ analysis was not required.

**Figure 4:**
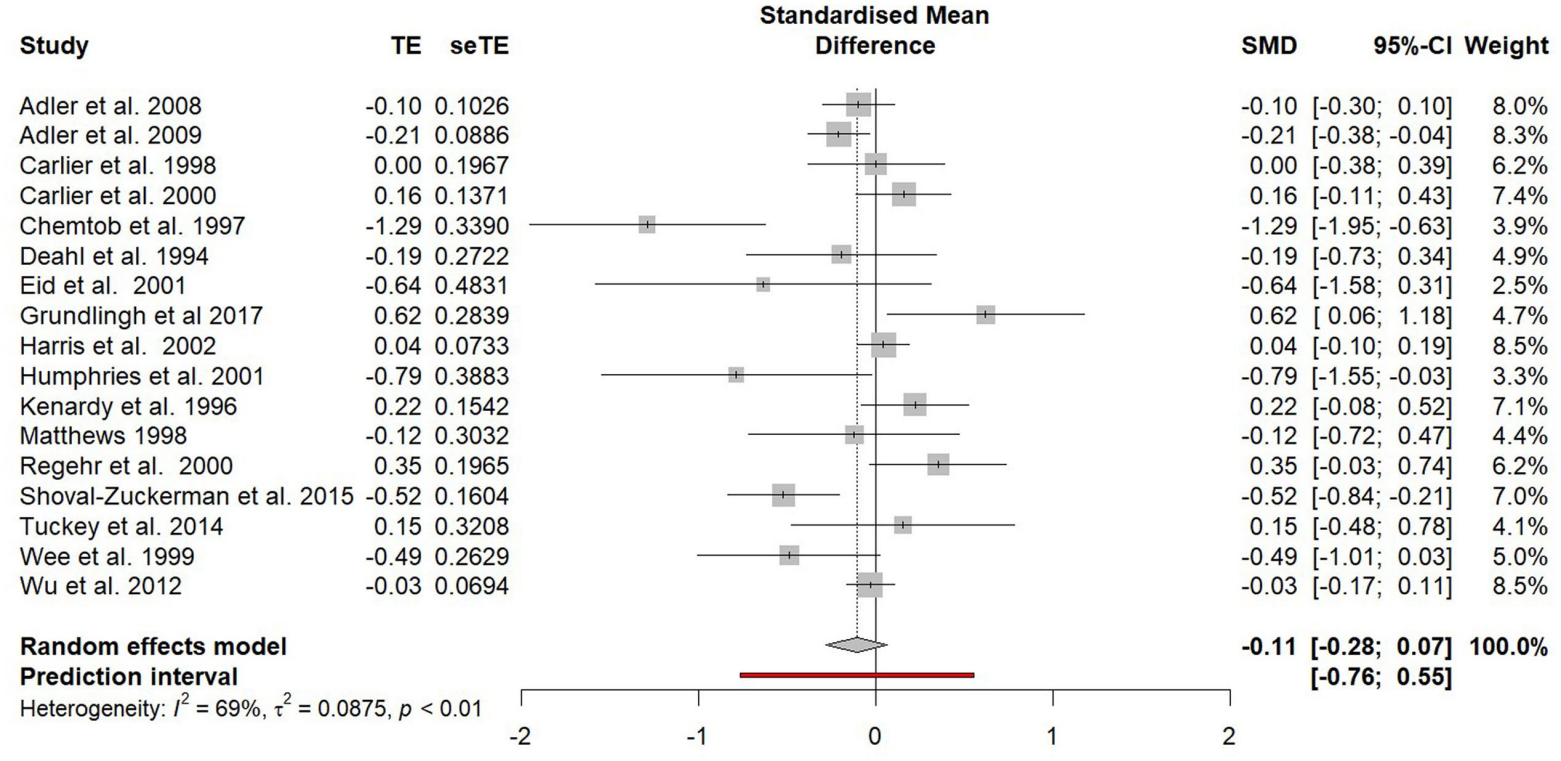
Forest plot of the standardised mean difference of PTSD symptoms between participants who did and did not receive psychological debriefing following a potentially traumatic event.

The random effects model was recalculated following the removal of the before-and-after studies and the combining of both the non-randomised controlled trials and randomised controlled trials (see Figure 4). An overall effect favouring psychological debriefing was found (SMD = −0.11). However, this effect was statistically non-significant (−0.28 to 0.07).

### The impact of time on effect size

**Figure 5:**
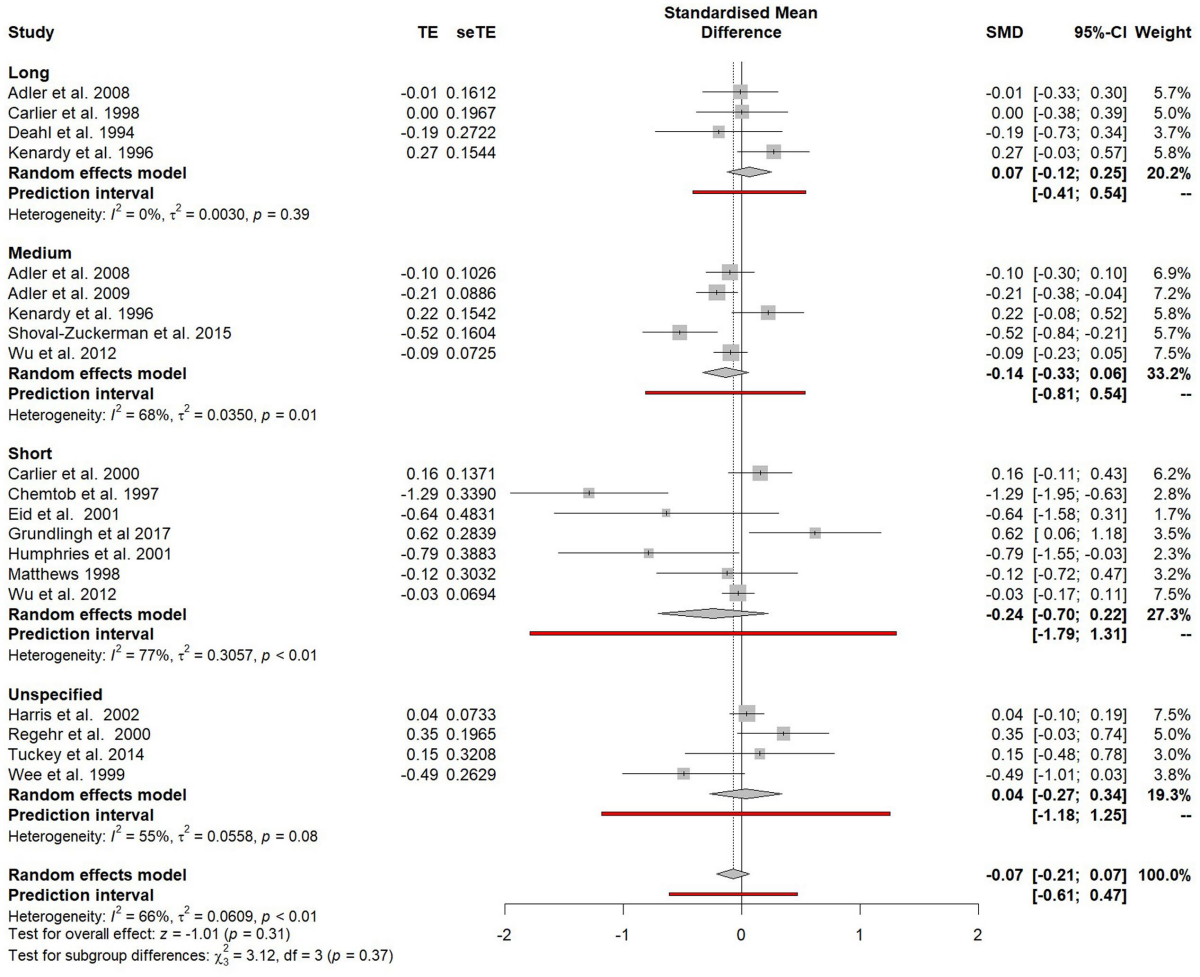
Forest plot of the standardised mean difference of PTSD symptoms between participants who did and did not receive psychological debriefing at different timepoints after debriefing.

To examine the impact of time of time on estimate effect size, a subgroup analysis was conducted to compare studies which collected short-term outcomes (0–3 months after debriefing), medium-term outcomes (4–6 months after debriefing) and long-term outcomes (7 months or more after debriefing; see Figure 5). For short-term outcomes, an effect favouring the intervention was reported (SMD = −0.24), but this effect was statistically non-significant (95% CI −0.70 to 0.22). For medium-term outcomes, an effect favouring the intervention was reported (SMD = −0.14), but again this was non-significant (CI −0.33 to 0.06). For long-term outcomes, a treatment effect close to zero was observed, although this did favour non-intervention (SMD = 0.07, 95% CI −0.12 to 0.25). Four studies could not be included in these subgroup comparisons because the timeframe between the PTE and outcome collected was either unspecified or varied between participants. As there was no significant difference between these subgroups (*χ*^2^ = 3.12, *p* = 0.37), they were combined for all subsequent analyses.

### The impact of risk of bias in the primary studies

To assess the impact of study-level risk of bias upon heterogeneity, a series of subgroup analyses were conducted on the estimates of SMD for the risk of bias ratings of “low risk” and “any risk” (unclear risk and high risk of bias combined) for each of the seven domains of methodological bias (see Table 3). No statistically significant differences in effect size estimates between studies with “low risk” of bias and “any risk” of bias were observed in any of the seven domains.

**Table 3:** Standard mean differences for studies with a “low risk” of bias and studies with “any risk” of bias within each of the seven risk domains.

	Low risk			Any risk				
	EFFECT	95% CI	*k*	EFFECT	95% CI	*k*	X^2^	*P*
Short term								
Selection bias	−0.08	−0.28 to 0.11	7	−0.19	−0.49 to 0.11	10	0.35	0.55
Performance bias	–	–	–	−0.11	−0.28 to 0.07	17	–	–
Treatment fidelity	−0.10	−0.20 to 0.01	4	−0.14	−0.40 to 0.11	13	0.12	0.73
Detection bias	−0.07	−0.27 to 0.13	13	−0.23	−0.59 to 0.13	4	0.58	0.45
Statistical bias	−0.11	−0.52 to 0.29	6	−0.10	−0.28 to 0.07	11	<0.01	0.96
Reporting bias	−0.14	−0.35 to 0.06	15	0.11	−0.11 to 0.33	2	2.66	0.10
Generalisability bias	−0.02	−0.14 to 0.09	8	−0.25	−0.62 to 0.12	9	1.35	0.25

### Differences in avoidance, hyperarousal and intrusion symptom outcomes

**Figure 6:**
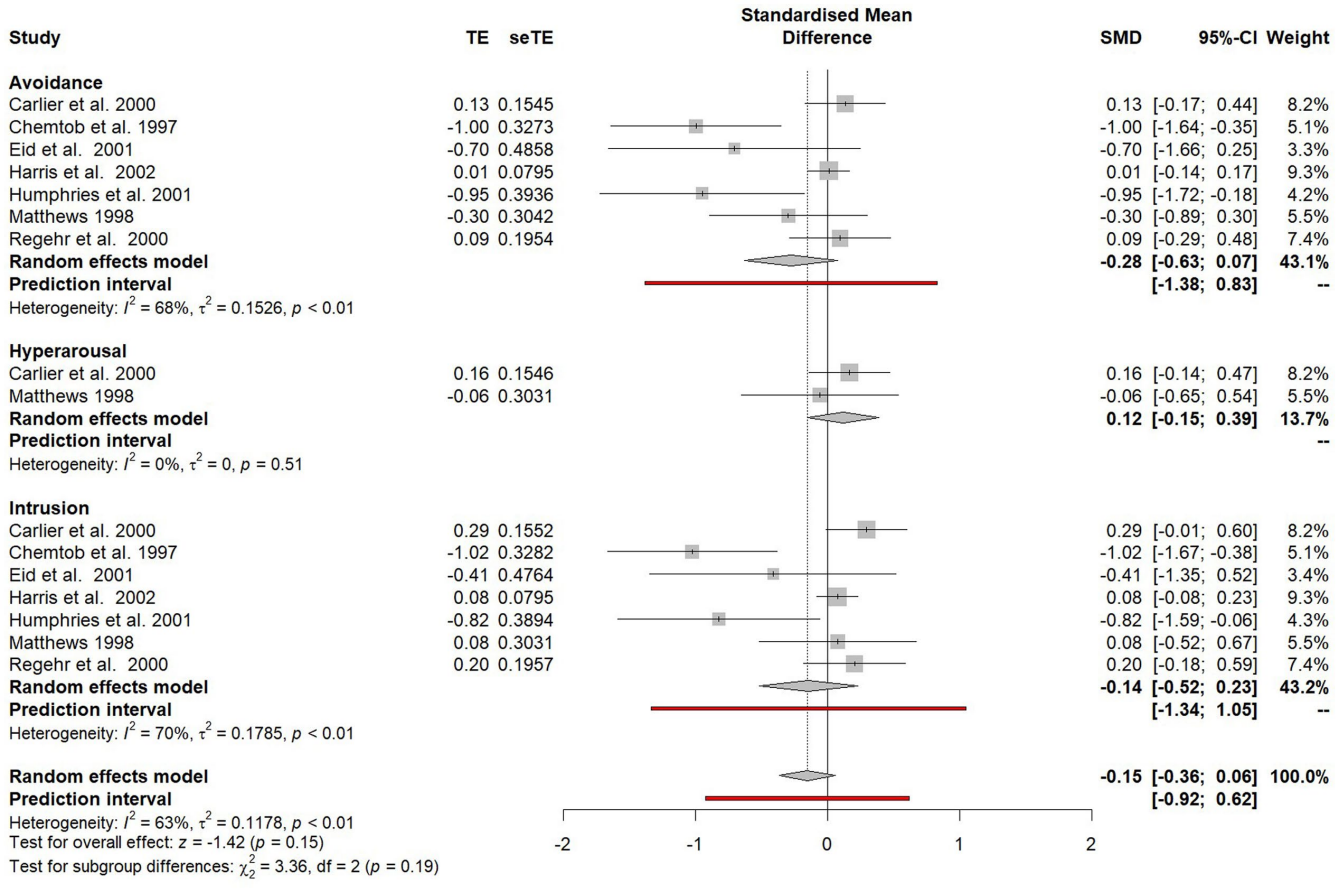
Forest plot of the standardised mean difference of specific PTSD symptom clusters between participants who did and did not receive psychological debriefing following a potentially traumatic event.

Outcomes were grouped in the three PTSD symptom clusters: avoidance, hyperarousal and intrusion. Studies which only reported total PTSD scores were excluded from this subgroup analysis. The difference between avoidance, arousal and intrusion symptoms was assessed in the subgroup plot shown in Figure 6.

No significant difference was found between outcomes on the three symptoms clusters (*χ*^2^ = 3.36, *p* = 0.19) and no significant treatment effects were observed for avoidance symptoms (SMD = −0.28, 95% CI 0.63–0.07), hyperarousal (SMD = 0.12, 95% CI −0.15 to 0.39) or intrusion symptoms (SMD = −0.14, 95% CI −0.52 to 0.23).

### Difference attributable to characteristics of psychological debriefings

#### Adherence to an established model of psychological debriefing

**Figure 7:**
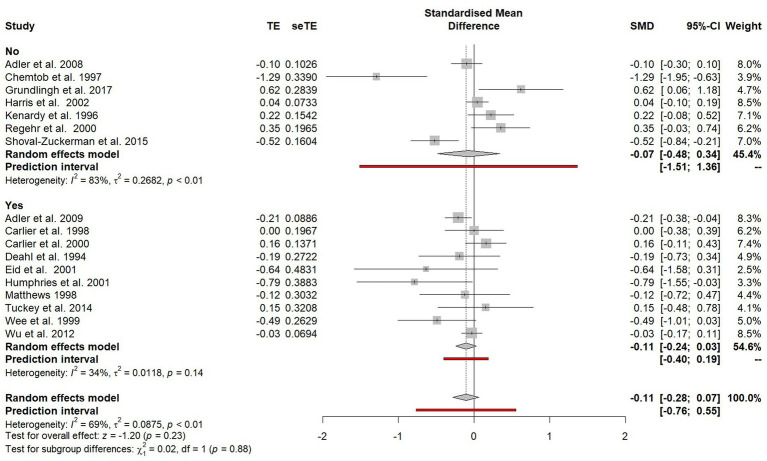
Subgroup plot of differences between studies that did and did not adhere to established seven phase models.

Studies were categorised according to whether or not assurances were given that the psychological debriefing adhered to the seven phase models outlined by Mitchell (1983), Dyregrov (1989), and Figure 7. There was no significant difference observed between those who did and did not adhere to the models (*χ*^2^ = 0.02, *p* = 0.88), although there was markedly less heterogeneity between studies that adhered to a seven phase model (*I*^2^ = 34%, *p* = 0.14) compared to those who did not (*I*^2^ = 83%, *p* < 0.01).

#### Single or multiple session debriefings

**Figure 8:**
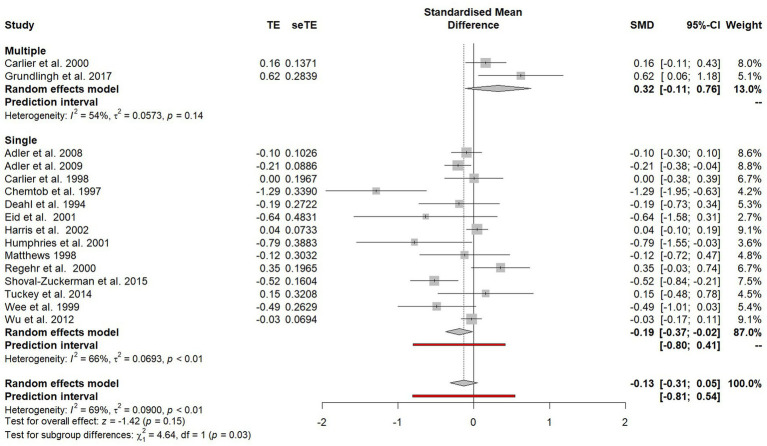
Subgroup plot of differences between studies offered single session debriefing versus those that offered multiple debriefing sessions.

Studies that evaluated a single-session debriefing were compared with studies that provided multiple debriefing sessions (see Figure 8). Kenardy et al. (1996) included data from both single and multiple session debriefings, so was excluded from this subgroup analysis. A significant difference (*χ*^2^ = 4.64, *p* = 0.03) favouring single session debriefing was observed and when only single-session debriefings were included in the analysis, a significant effect was found (SMD = −0.19, 95% CI −0.37 to −0.02).

#### Individual or group debriefings

**Figure 9:**
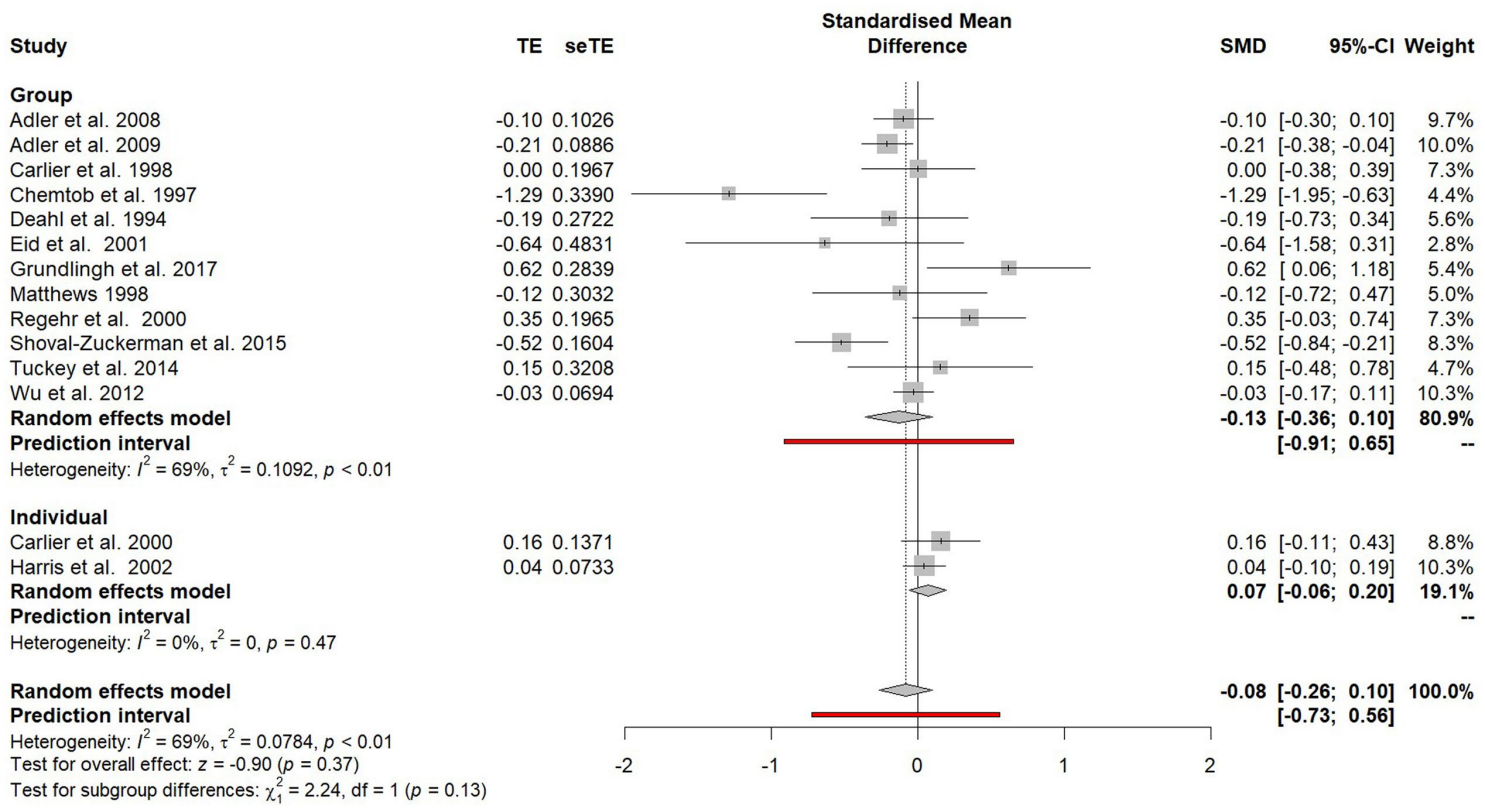
Subgroup plot of differences between individual and group debriefings.

Studies that evaluated group debriefings were compared with studies that evaluated individual debriefings (see Figure 9). Three studies did not specify whether debriefings were done with groups or individuals (Kenardy et al., 1996; Wee et al., 1999; Humphries and Carr, 2001) and so they were excluded from this subgroup analysis. No statistically significant difference was observed between the two subgroups (*χ*^2^ = 2.24, *p* = 0.13).

### Differences attributable to trauma characteristics

#### Single or multiple traumatic incidences

**Figure 10:**
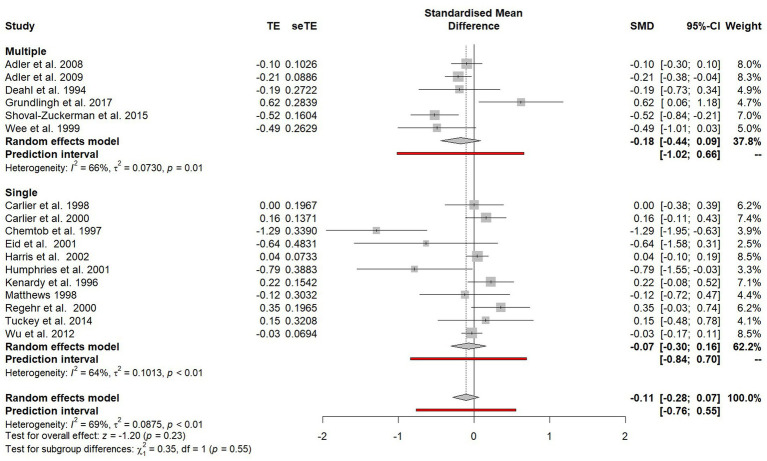
Subgroup plot of differences between single or multiple traumatic incidences.

Outcomes were compared for participants who had experienced a single PTE versus participants who were reported to have experienced multiple PTEs (see Figure 10). There was no significant difference observed in effect sizes between participants exposed to a single PTE and participants exposed to multiple PTEs (*χ*^2^ = 0.35, *p* = 0.55).

### Subgroup analyses that were not possible to conduct

Data was organised so that subgroup analyses could also be conducted in other areas including the length of debriefing, the length of time between the PTE and debriefing and the extent of debriefers training. However, in several of the studies, this information was not reported and so these subgroup analyses could not be conducted.

### Publication bias and small-study effects

Small-study effects refers to the tendency for studies with smaller sample sizes to show different and often larger treatment effects than studies with larger sample sizes (Rücker et al., 2011). One possible reason for this is publication bias, whereby statistically significant results are more likely to be published than non-significant results (Rothstein et al., 2006). Firstly, in smaller studies, larger treatment effects are needed for a result to be statistically significant. Secondly, due to the higher levels of resource and often higher methodological quality of larger studies, non-significant results in larger studies are more likely to be published than non-significant results in smaller studies (Sterne et al., 2000).

Bias in a meta-analysis may be assessed visually using a funnel plot; a simple scatter plot of the treatment effect estimates from each primary study against a measure of study size (Rothstein et al., 2006). If there is an absence of bias, the plot will resemble a symmetrical inverted funnel as the effects from the smaller studies at the bottom of the plot show greater variability than the larger studies at the top of the plot, which will lie closer to the overall meta-analytic effect. However, if there is an absence of studies in the area of the plot associated with small sample sizes and non-significant results, it is likely that publication bias is resulting in an overestimation of the true effect size.

**Figure 11:**
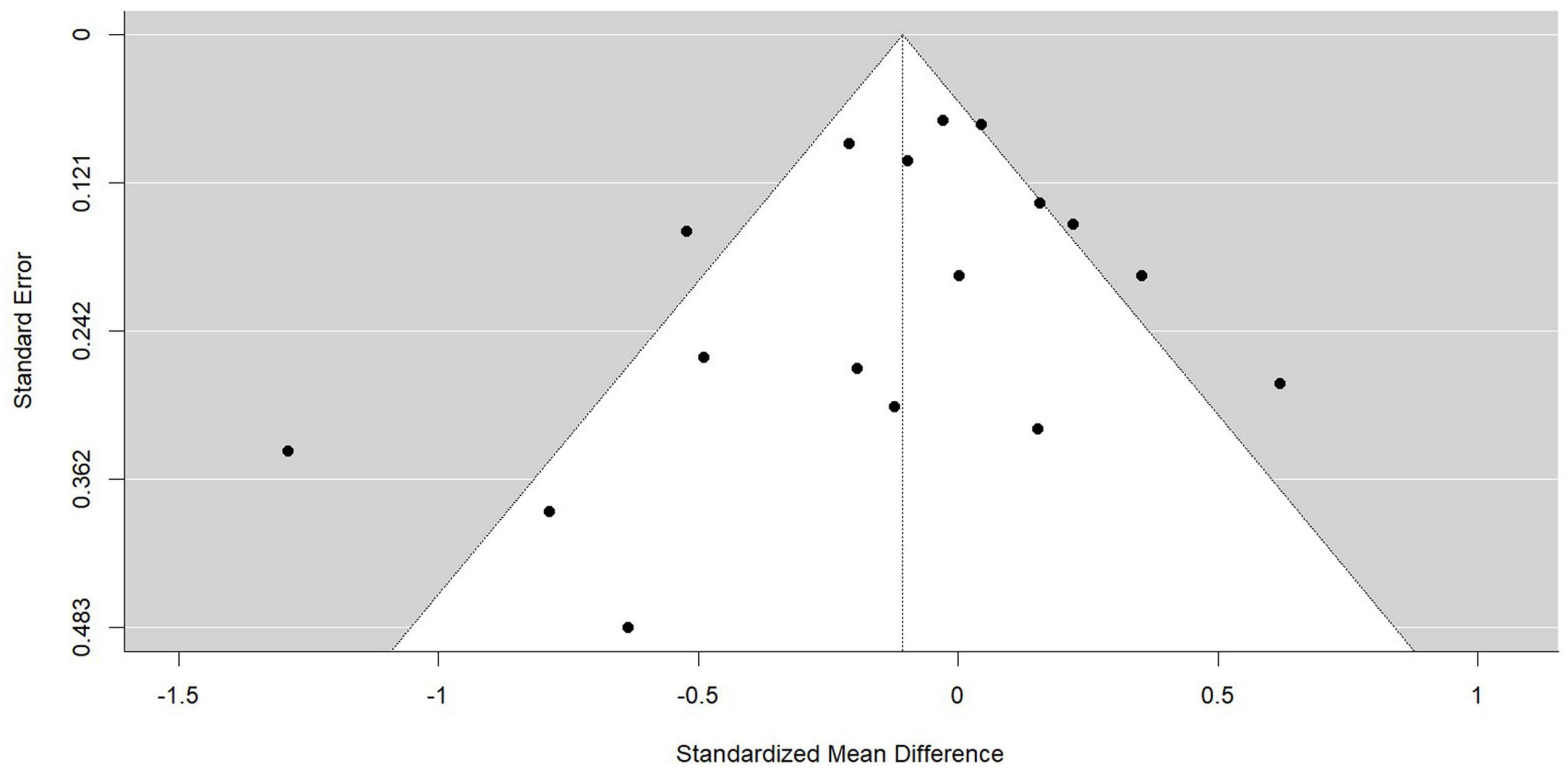
Funnel plot of the standardised mean difference for all PTSD symptom outcomes. The 95% confidence interval of the expected distribution of treatment effects is shown as an inverted ‘funnel’. The highlighted area in blue is that associated with publication bias.

Visual inspection of the funnel plot (see Figure 11) would suggest the presence of publication bias as there appears to be an absence of studies with higher standard errors (i.e., smaller samples) around the area of the funnel plot consistent with null results (standardised mean difference = 0). In addition, the heterogeneity of this data is evident in the number of SMD outside of the expected 95% confidence interval.

The trim-and-fill method (Duval and Tweedie, 2000a,b) was used to detect and adjust for the publication bias evident in the funnel plots asymmetry. The trim-and-fill method involves iteratively removing the most extreme small studies from the positive side of the funnel plot and re-computing the effect size at each iteration until the funnel plot is symmetrical about a corrected effect size. The omitted studies are then added back into the analysis and a mirror image for each of these studies is imputed. The trim-and-fill procedure did not identify statistically significant funnel plot asymmetry and therefore did not result in any corrections to the current analysis.

## Discussion

The aim of this meta-analysis was to evaluate the effectiveness of psychological debriefing in preventing or reducing PTSD symptoms following a work-related PTE and identify factors that appear to impact the effectiveness of psychological debriefing though a series of subgroup analyses.

It was recognised that the unpredictable nature of trauma means that most trauma research cannot meet the gold-standard of study design insisted upon by the Cochrane Library and so a variety of study designs were initially included within this study, including uncontrolled before-and-after studies or studies which lacked a suitable control group so were treated as before-and-after studies. However, in the absence of a control group in these studies, it was not possible to determine whether psychological debriefing resulted in an improvement over and beyond that of natural recovery and the particularly high effect sizes in these studies suggested that maturational effects and other potential biases influenced outcomes. Consequently, little could be inferred from these studies’ results, and they were removed from the meta-analysis.

While four of the controlled studies included in the meta-analysis found a statistically significant positive effect of psychological debriefing, with only one finding a significant negative effect (Grundlingh et al., 2017), the overall synthesis did not find consistent and substantive evidence that psychological debriefing helps to prevent or reduce PTSD symptoms following a work-related PTE. In their paper, Grundlingh et al. (2017) suggest that the lower levels of traumatic stress (as measured by the IES-R) within the control group may have been due to a lack of self-awareness or minimisation of trauma reactions. In contrast, due to the psychoeducational nature of the debrief sessions, the relatively higher levels of reported traumatic stress in the intervention group may be due to heightened awareness. It is also of note that this was the only study that involved individuals exposed to PTE vicariously (researchers interviewing children in Uganda who had experienced violence) and the research also found no evidence of elevated emotional distress in the violence researchers following these interviews, suggesting that psychological debriefing was not indicated. Nevertheless, perceived organisational support was associated with lower levels of distress, highlighting the importance of support structures being in place.

Only one of the subgroup analyses conducted produced a statistically significant finding. Single-session debriefings were found to produce better outcomes than multiple-session debriefings. Furthermore, when analysis was limited to studies that solely evaluated single-session debriefing, a significant effect favouring psychological debriefing was found. This result contrasts with the non-significant finding of the Cochrane review (Rose et al., 2002) which only evaluated single-session psychological debriefings. It is important to note that heterogeneity in outcomes between studies was high and, while the overall effect was significant, the overall effect size was small (*d* = −0.19; Cohen, 1992). Nevertheless, the finding brings into question the assertion that “single-session debriefing may be at best ineffective” (NICE, 2005, p. 84).

### Recommendations for future research and service providers

While heterogeneity of the controlled trials was below the pre-defined 75% threshold, there was still substantial variation between studies. The subgroup analysis on adherence to the seven phase models outlined by Mitchell (1983) and Dyregrov (1989) explains much of this variation. There was markedly less heterogeneity in effect sizes between the studies which adhered to a seven phase model compared to the studies that evaluated interventions referred to as psychological debriefing but that were either significantly modified or did not offer any assurances a standardised seven phase model was used. The apparent confusion and inconsistency in the literature regarding use of the term ‘psychological debriefing’ has been previously recognised (Tuckey, 2007). This lack of clarity has hampered research progress and increased the likelihood of misapplication of research findings. Future research in this area should ensure that the psychological debriefing being evaluated adheres to an established standardised seven phase model. This will improve the robustness of the evidence base in this field.

Many other subgroup analyses were unable to be conducted due to unreported information within studies, including some directly linked to the recommendations made about psychological debriefing by Hawker and Hawker (2015). These included the impact of the timing of psychological debriefing following a PTE, the length of debriefing sessions and the qualifications and training of facilitators. For some studies, the absence of this information was simply due to poor reporting. For other studies, it was due to methodological shortcomings. For example, the four studies relying on naturalistic methods (Kenardy et al., 1996; Wee et al., 1999; Regehr and Hill, 2001; Harris et al., 2002) had no influence on the provision of debriefings and only limited knowledge about the nature of the interventions they were evaluating.

Tuckey (2007) makes a number of recommendations regarding the clarity of reporting in psychological debriefing research. These recommendations include clearly and accurately reporting the level of training and experience of the debriefers, the timing of the debriefing sessions relative to the potentially traumatic events, and the size of the group debriefing sessions. Following these recommendations would, again, improve the robustness of the evidence base into the effectiveness of psychological debriefing.

While recommendations have been made to improve the robustness of the evidence base, it appears that research into psychological debriefing has reduced in recent years. Most of the 21 articles included within this meta-analysis were published before or around the turn of the millennia, with only seven published since 2002 when the Cochrane review on psychological debriefing (Rose et al., 2002) was published. Hawker et al. (2011) note the difficulties in obtaining ethical approval and funding for research in this area in the present day due to the widespread belief that psychological debriefing is harmful. Yet this meta-analysis suggests that future studies, which both adhere to a standardised model and clearly and accurately report on the nature of the psychological debriefing being offered, are warranted.

It is hoped that future NICE guidance will encourage further research in psychological debriefing for groups of trauma-exposed staff and a more robust evidence base will follow. Until then, for those organisations who are continuing to provide psychological debriefing, there are several recommendations stemming from this meta-analysis and the reviews into psychological debriefing which have preceded it (Hawker and Hawker, 2015; Richins et al., 2020): psychological debriefing should be optional rather than compulsory, delivered by trained facilitators, adhere to an established model while also being informed by the organisational culture, and have the support of management.

### Limitations

There are some limitations to this meta-analysis which must be acknowledged. There were significant methodological shortcomings in many of the studies included in the synthesis. As previously noted, some studies had no control over the nature of the psychological debriefings provided to participants. In other studies, attrition rates were very high. One of the most noticeable methodological limitations to several of the studies related to the recruitment of control groups. In four studies, control groups were taken from a different occupational group or geographical area. In two studies, intervention and control groups were formed through self-selection, with the control group comprising of those who had declined debriefing. These approaches are likely to have introduced selection bias.

There are inherent difficulties in establishing appropriate control groups within trauma research. It is important that psychological debriefing is optional rather than mandatory and, conversely, that available interventions are not intentionally withheld from people. Furthermore, given the early nature of the intervention, waiting-list control groups are often not practicable. Consequently, it is to be expected that studies resort to self-selection methods to form intervention and control groups. However, in these cases it is important baseline assessments are administered to ensure there is no differences in symptom severity between the groups prior to intervention or, if there is, that this is accounted for using an interaction effect between group and time.

A second limitation is that all but two of the studies included relied on self-report outcome measures. While subjective experience of symptomology is important, the psychoeducational component of debriefing may have increased participant’s awareness of symptoms and, therefore, increased their self-reported scores on outcome measures (Grundlingh et al., 2017).

A third limitation is that outcomes were restricted to PTSD symptoms. While the addition of further outcomes would have resulted in an unwieldy analysis, Richins et al. (2020) note that additional outcome measures in primary studies may uncover other benefits. For example, Tuckey and Scott (2014) found that emergency service personnel who had been debriefed following a PTE consumed less alcohol as a means of coping and reported better quality of life. Furthermore, Richins et al. (2020) note the high proportion of studies evaluating group-based early interventions where peer support was reported to facilitate recovery or improve experience. These identified social benefits of psychological debriefing may not be captured by measures of PTSD symptoms but could still make psychological debriefing a worthwhile intervention.

Fourthly, while the meta-analysis focused specifically on work-related PTEs, the scope of studies included was still large. One of the greatest variations between studies was the length of time between a PTE and the psychological debriefing. This ranged from 24 h (Carlier et al., 2000) to 6 months (Chemtob et al., 1997). Mitchell and Everly (1996 p. 87) only caution against the use of psychological debriefing “several months” after a PTE and so this timeframe does not necessarily go against established recommendations. Nevertheless, interventions at different timeframes are likely to serve different functions. Unfortunately, it was not possible to explore the impact of timing of psychological debriefing on PTSD symptomology and so studies using markedly different timeframes were combined throughout the analysis.

Finally, though this meta-analysis sought to be methodologically robust through the application of guidance provided by PRISMA (Borenstein et al., 2009; Page et al., 2021), all data extraction was completed by one review author. The Cochrane Library recommend that more than one person extracts data to minimise errors and the risk of bias being introduced by review authors (Higgins et al., 2019).

## Conclusion

It appears that, for now, the debriefing debate will continue. While the overall synthesis in this meta-analysis did not provide any consistent and substantive evidence that psychological debriefing improves natural psychological recovery after a traumatic event, the findings also suggest that Rose et al.’s (2002) conclusion that “psychological debriefing is either equivalent to, or worse than, control or educational interventions in preventing or reducing the severity of PTSD” (p. 2) may have been premature. The widespread belief that psychological debriefing is harmful appears to have hindered the progress of research in this field. It is hoped that further well-designed studies that account for the methodological limitations inevitable in trauma research are conducted. This will help to inform organisations’ provision of intervention following work-related PTEs and ultimately ensure that employees receive the effective support they need and deserve.

## Data availability statement

The original contributions presented in the study are included in the article/Supplementary material, further inquiries can be directed to the corresponding author.

## Author contributions

HS submitted this meta-analysis as a chapter for his ClinPsyD thesis, completing the literature review, data analysis and write up. CJ was HS’s research supervisor, supporting significantly with the data extraction and data analysis and providing feedback on this write up. All authors contributed to the article and approved the submitted version.
