# Supplementary material for: Revisiting the debriefing debate: does psychological debriefing reduce PTSD symptomology following work-related trauma? A meta-analysis
Source: Front Psychol. 2023 Dec 21;14:1248924. doi: 10.3389/fpsyg.2023.1248924 (PMC10779682; doi:10.3389/fpsyg.2023.1248924)
Supplement: Supplementary file 1 [file Table_1.docx]

**Supplementary Table 1.** Study design hierarchy.

| **Study Design** | **Quality Score** | **Description** |
| --- | --- | --- |
| Randomised controlled trial/experiment (including cluster randomisation) | 30 | An experimental study comparing two (or more) groups to establish the effectiveness of a specific intervention. An experimental group receives the intervention, while a comparison or control group receives either an alternative intervention or no intervention. Participants (or groups of participants) are randomly assigned to a group to minimise bias. |
| Non-randomised controlled trial/experiment | 20 | An experimental study in which people are allocated to either experimental or comparison/control groups using methods that are not random. As a result, there is an increased risk of biases being introduced into the research. |
| Repeated measures design (before-and-after studies without a separate control group) | 10 | A study in which observations are made before and after the implementation of an intervention. Data is collected at baseline and one or more times after the procedure. Uncontrolled before-and-after studies are an intrinsically weak evaluative design as they are unable to rule out alternative explanations for observed effects. |
