# Supplementary material for: Revisiting the debriefing debate: does psychological debriefing reduce PTSD symptomology following work-related trauma? A meta-analysis
Source: Front Psychol. 2023 Dec 21;14:1248924. doi: 10.3389/fpsyg.2023.1248924 (PMC10779682; doi:10.3389/fpsyg.2023.1248924)
Supplement: Supplementary file 2 [file Table_2.docx]

**Supplementary Table 2.** Criteria for ratings of low, unclear or high risk across seven domains.

| **Domain** | **Details** | **Low risk of bias** | **Unclear risk of bias** | **High risk of bias** |
| --- | --- | --- | --- | --- |
| **Selection Bias** | Systematic differences between baseline characteristics of the groups that are compared. | Non-response rate is reported and of an acceptable level (< 50%).  The source population is well described, and the study reports the characteristics of the sample e.g. the study details subgroups.   The recruitment method is clearly reported and well defined.  The article provides some reassurance that there is no selection bias (e.g. allocation concealment). | Non-response rate is not reported.  The characteristics of the study population are not clearly reported. For example, the country, setting, location, population demographics are not adequately reported.   The recruitment process/ sampling method of individuals are unclear or has not been reported. | Non-response rate is at unacceptable level (>50%)  There are clear differences between groups being compared (e.g. experimental and control arms are from different populations).  The characteristics of the study population are not reported. |
| **Performance Bias** | Systematic differences between/within groups in the participants motivation to complete the study or in exposure to factors other than the interventions of interest. | Study reports level of confidentiality and anonymity.  Participants were not rewarded for their participation in the study.  Information and procedures are provided in a way that does not differentially motivate participants.  Participants were blinded where self-report measures are used. | The study does not report levels of confidentiality and anonymity.  It is not clear if participants were rewarded for their participation.  It is unclear how much information was provided to the participant prior to taking part in the study.  Self-report measures are used but there is no evidence that participants were blinded. | Responses are not confidential or anonymous.  Participants were rewarded for their participation in the study. |
| **Treatment Fidelity** | The extent to which the treatment is delivered competently and as intended and is representative of the class of treatments to which the study intends to generalise. | Treatment is sufficiently well described that it could be replicated.   Treatment corresponded to intended treatment described in the methodology and established psychological debriefing protocols  Procedures were in place to assess the fidelity of administered treatment. | Treatment protocol is unclear or has not been reported.  There is no evidence that procedures are in place to assess the fidelity of administered intervention. | The treatment provided was different than the intended treatment.  Treatment is provided inconsistently between participants.  Treatment is not in line with established psychological debriefing protocols. |

| **Domain** | **Details** | **Low risk of bias** | **Unclear risk of bias** | **High risk of bias** |
| --- | --- | --- | --- | --- |
| **Detection Bias** | Systematic differences between participants in how outcomes are determined.  The extent to which the study design is optimised to detect the effect in questions. | The outcome measures are clearly defined, valid and reliable, and are implemented consistently across all participants.  Outcomes were blindly rated by assessors (when an alternative to self-report measures have been used). | Information regarding the outcome measures are either not reported or not clearly reported e.g. definition, validity, reliability.  The outcome measure(s) used has questionable psychometric properties (e.g. Cronbach's Alpha is between 0.6 and 0.7)  It is not clear if the measure was implemented consistently across all participants. | The outcome measures were implemented differently across participants.  The outcome measures used had poor reliability and validity reported e.g Cronbach’s Alpha < 0.6.   Only one dimension/subscale of the scale is used. |
| **Statistical Bias** | Bias resulting from the inappropriate statistical treatment of the data. This includes using completer-only analysis rather than intention-to-treat or other methods for inputting missing data. | Appropriate statistical testing was used.  Confidence intervals or exact p-values for effect estimates were given or possible to calculate.  Attrition rate – data loss is reported at analysis at an acceptable level (<5%) and appropriate method is used for inputting missing data. | It is unclear what statistical test was used.  Confidence intervals or exact p-values for effect estimates were not reported and could not be calculated.  Attrition rate – data loss is not reported at analysis or is at 10-20% | Statistics were not reported.  Wrong statistical test was used which was not appropriate for the study design.  Attrition rate – data loss is reported at analysis at an unacceptable level (>30%) |
| **Reporting Bias** | Systematic differences between reported and unreported findings (e.g., selective reporting of statistically significant findings). | Study has reported all results of measures as outlined in the method.   Reasons for attrition or exclusions are reported. | Not all descriptive and/or summary statistics are presented.  There is a description (narrative) in the results, but statistics are not recorded. | Study has not reported full outcome measures that are stated in the method section/ reported only a subsample of results/only significant results/ not reported the measure as it should be. |
| **Generalisation** | The extent to which the sample represents the target population from which it was drawn. | Sufficient sample size (35+ per arm) and representative of target population.  A sample size justification, estimate or power analysis is provided. | 20-30 participants per arm.  Idiosyncratic features in sample.  A sample size justification, estimate or power analysis are not provided. | Small sample (10-20 per arm) with or without idiosyncratic feature.  Sample is not representative of wider profession. |
