# Supplementary material for: Revisiting the debriefing debate: does psychological debriefing reduce PTSD symptomology following work-related trauma? A meta-analysis
Source: Front Psychol. 2023 Dec 21;14:1248924. doi: 10.3389/fpsyg.2023.1248924 (PMC10779682; doi:10.3389/fpsyg.2023.1248924)
Supplement: Supplementary file 3 [file Table_3.docx]

**Supplementary Table 3.** Ratings of risk of bias. Red indicates high risk of bias, amber marks an unclear risk of bias and green is a low risk of bias.

| ***Study*** | ***Study Design*** | ***Selection Bias*** | ***Performance Bias*** | ***Treatment Fidelity*** | ***Detection Bias*** | ***Statistical Bias*** | ***Reporting Bias*** | ***Generalisability*** | ***Quality Index*** |
| --- | --- | --- | --- | --- | --- | --- | --- | --- | --- |
| Adler et al. (2008) | Randomised controlled trial/experiment | Low risk | Unclear risk | Low risk | Low risk | High risk | Low risk | Low risk | 93% |
| Adler et al. (2009) | Randomised controlled trial/experiment | Low risk | Unclear risk | Low risk | Low risk | High risk | Low risk | Low risk | 93% |
| Campfield et al. (2001) | Before-and-after study | Low risk | Unclear risk | Unclear risk | Low risk | Low risk | Low risk | High risk | 24% |
| Carlier et al. (1998) | Non-randomised controlled trial/experiment | Low risk | Unclear risk | Unclear risk | Low risk | Unclear risk | Unclear risk | Low risk | 68% |
| Carlier et al. (2000) | Non-randomised controlled trial/experiment | High risk | Unclear risk | Unclear risk | Unclear risk | Unclear risk | High risk | Unclear risk | 57% |
| Chemtob et al. (1997) | Non-randomised controlled trial/experiment | High risk | Unclear risk | High risk | Low risk | Unclear risk | Low risk | High risk | 59% |
| Deahl et al. (1994) | Non-randomised controlled trial/experiment | Unclear risk | Unclear risk | Unclear risk | Low risk | Unclear risk | Low risk | Unclear risk | 66% |
| Eid et al. (2001) | Non-randomised controlled trial/experiment | High risk | Unclear risk | Unclear risk | Low risk | Low risk | Low risk | High risk | 64% |
| Grundlingh et al. (2017) | Randomised controlled trial/experiment | Low risk | Unclear risk | Unclear risk | Low risk | Low risk | Low risk | Unclear risk | 93% |
| Harris et al. (2002) | Non-randomised controlled trial/experiment | Unclear risk | Unclear risk | High risk | Low risk | Low risk | Low risk | Low risk | 68% |
| Humphries & Carr (2001) | Non-randomised controlled trial/experiment | High risk | Unclear risk | Unclear risk | Low risk | Low risk | Low risk | High risk | 64% |
| Kenardy et al. (1996) | Non-randomised controlled trial/experiment | Unclear risk | Unclear risk | High risk | Low risk | Unclear risk | Low risk | Low risk | 66% |
| Matthews (1998) | Non-randomised controlled trial/experiment | High risk | Unclear risk | Unclear risk | Unclear risk | High risk | Low risk | High risk | 57% |
| Regehr & Hill (2000) | Non-randomised controlled trial/experiment | Unclear risk | Unclear risk | High risk | Low risk | Low risk | Low risk | Low risk | 68% |
| Richards (2001) | Before-and-after study | Low risk | Unclear risk | Unclear risk | Low risk | High risk | Low risk | Low risk | 45% |
| Ruck et al. (2013) | Before-and-after study | High risk | Unclear risk | Unclear risk | Low risk | High risk | Low risk | Low risk | 41% |
| Shoval-Zuckerman et al. (2015) | Non-randomised controlled trial/experiment | Low risk | Unclear risk | Unclear risk | Unclear risk | Low risk | Low risk | Unclear risk | 68% |
| Tehrani et al. (2001) | Before-and-after study | Low risk | Unclear risk | High risk | Unclear risk | Low risk | Low risk | High risk | 21% |
| Tuckey et al. (2014) | Randomised controlled trial/experiment | Low risk | Unclear risk | Unclear risk | Low risk | High risk | Low risk | Unclear risk | 89% |
| Wee et al. (1999) | Non-randomised controlled trial/experiment | Unclear risk | Unclear risk | High risk | Unclear risk | Unclear risk | Low risk | Low risk | 64% |
| Wu et al. (2012) | Randomised controlled trial/experiment | Low risk | Unclear risk | Low risk | Low risk | Unclear risk | Low risk | Low risk | 95% |
